# Cutaneous Aspergillosis Among Extremely Preterm Neonates in Neonatal Intensive Care Units — Michigan and New York, 2023–2024

**DOI:** 10.15585/mmwr.mm7543a2

**Published:** 2026-11-05

**Authors:** Ramya Naraharisetti, Daniel Baker, Jevon McFadden, Tina France, Monica Quinn, Joseph Coyle, Ernest Clement, Brenda Brennan, Sudha Chaturvedi, Jennifer Jones, Gabrielle C. Todd, Denise Parr, Mitsuru Toda, Nancy Chow, Daliya Khuon, Mallory Davis, Jessica McClusky, Maggie Simons, Gabriel Laney, Alex George, Steve Moore, Brianne Bachman, Erika Kurili, Christopher Prestel, Elizabeth T. Sajewski, Jeremy A.W. Gold

**Affiliations:** ^1^Epidemic Intelligence Service, CDC; ^2^Bureau of Infectious Disease Prevention, Michigan Department of Health and Human Services; ^3^New York State Department of Health; ^4^U.S. Public Health Service; ^5^Division of State and Local Readiness, Office of Readiness and Response, CDC; ^6^Wadsworth Center, New York State Department of Health; ^7^Corewell Health, Grand Rapids, Michigan; ^8^Division of Foodborne, Waterborne, and Environmental Diseases, National Center for Emerging and Zoonotic Infectious Diseases, CDC; ^9^Division of Healthcare Quality Promotion, National Center for Emerging and Zoonotic Infectious Diseases, CDC.

**Summary**

**What is already known about this topic?**

Cutaneous aspergillosis is a rare, life-threatening infection in neonates, particularly in extremely preterm infants (<28 weeks’ gestational age).

**What is added by this report?**

During 2023–2024, two cutaneous aspergillosis clusters were detected in neonatal intensive care units in Michigan (two patients, including one death) and New York (three patients, no deaths). No definitive source was identified; investigations prompted improvements in linen handling, air-handling measures, and infection prevention practices.

**What are the implications for public health practice?**

Clinicians should consider cutaneous aspergillosis in extremely preterm neonates with skin lesions. Early recognition and coordinated assessment by clinicians, infection preventionists, and facility engineers can guide mitigation, improve outcomes, and prevent additional cases.

## Abstract

Cutaneous aspergillosis is a rare, life-threatening fungal infection in neonates, particularly those born extremely preterm (gestational age <28 weeks); outbreaks have been linked to environmental sources of *Aspergillus* species. The Michigan Department of Health and Human Services and the New York State Department of Health investigated two clusters of cutaneous aspergillosis in neonatal intensive care units during 2023–2024. In Michigan, two extremely preterm neonates (gestational age <24 weeks) received a diagnosis of cutaneous aspergillosis. Samples cultured from both infants were positive for *Aspergillus fumigatus*; both received antifungal treatment, and one infant died. In New York, three neonates (two born at gestational age 24–25 weeks and one at <24 weeks) received a diagnosis of cutaneous aspergillosis. Isolates from skin lesions were identified as *A. fumigatus* (one isolate) and closely related *Aspergillus quadrilineatus* (two isolates); all infants received antifungal treatment, and all three survived. In Michigan, *A. fumigatus* was isolated from an air sample collected near linen-transport areas; environmental sampling was not performed in New York. No definitive environmental source was identified for either cluster. Both investigations led to targeted infection control interventions, including revised linen handling, optimized airflow and air-handling systems, and enhanced skin assessment and infection prevention practices. These clusters underscore the susceptibility of extremely preterm neonates to invasive mold infections and the importance of early recognition, thorough environmental assessment and multidisciplinary collaboration, to prevent further exposures.

## Introduction

Cutaneous aspergillosis is a rare, life-threatening infection caused by *Aspergillus* species, a group of ubiquitous environmental molds. Extremely preterm neonates^†^ are at increased risk for this infection because of their immature immune defenses, underdeveloped skin barriers, and frequent exposure to invasive medical devices (*1*). Previous health care–associated mold clusters and outbreaks, including neonatal intensive care unit (NICU) cutaneous aspergillosis clusters, have been linked to construction, contaminated health care textiles, contaminated medical equipment, and breaches in infection prevention and control (*2*–*5*). During October 2023, the Michigan Department of Health and Human Services was notified of two patients with cutaneous aspergillosis in a level IV^§^ NICU. During July–September 2024, the New York State Department of Health was notified of three patients with cutaneous aspergillosis in a separate level IV NICU.

## Investigation and Public Health Response

A case of cutaneous aspergillosis was defined as isolation of *Aspergillus* species from cultures of suspicious skin lesions (e.g., erythema or ulceration) from a patient admitted to the Michigan NICU during October–November 2023 or the New York NICU during July–September 2024. This activity was reviewed by CDC, deemed not research, and conducted consistent with applicable federal law and CDC policy.^¶^

### Michigan NICU

In Michigan, during October 2023, clinicians identified skin lesions at multiple sites on an extremely preterm neonate (gestational age [GA] <24 weeks) during the first week of life (Table). Cultures from skin lesions and biopsy specimens grew *Aspergillus fumigatus*, and species identification was confirmed by polymerase chain reaction (PCR) amplification and sequencing of the internal transcribed spacer (ITS) region, (*6*) followed by Basic Local Alignment Search Tool (BLAST) comparison of the resulting sequence with reference sequences. After identification of the first case, the facility implemented enhanced skin-assessment protocols, with a lower threshold for escalating findings to a health care provider. Approximately 2 weeks later, a second case of *A. fumigatus* infection was detected in another extremely preterm neonate (GA <24 weeks), also during the first week of life. The organism was isolated from a culture of a skin sample from a lesion on the infant’s trunk and confirmed by PCR. Both neonates received amphotericin B. The first infant died during the second week of life; the death was attributed to septic shock from aspergillosis, *Pseudomonas* species infection, and extreme prematurity. The Michigan investigation included on-site observations, staff member interviews, and environmental sampling; one air sample collected near a corridor used for linen transport grew *A. fumigatus*. The facility implemented multiple mitigation measures, including converting negative-pressure areas in the shared nursery to positive pressure to reduce the risk for airborne spore transmission; repairing plumbing leaks, carpeting, and flooring; and designating a separate loading dock for clean linens and supplies.

**TABLE:** Clinical characteristics, treatment, and outcomes of extremely preterm, extremely low birthweight neonates* with cutaneous aspergillosis in neonatal intensive care units — Michigan and New York, 2023–2024

Characteristic	State and patient				
	Michigan NICU		New York NICU		
	Patient A	Patient B	Patient C	Patient D	Patient E
Gestational age	<24 wks	<24 wks	24–25 wks	24–25 wks	<24 wks
Age at initial lesion detection	First week of life	First week of life	First week of life	First week of life	Second week of life
Lesion location	Multiple sites	Trunk	Back	Back	Back
***Aspergillus* species identification**					
By culture	*A. fumigatus*	*A. fumigatus*	*A. fumigatus*	*A. nidulans*	Non-*fumigatus Aspergillus* species
By molecular methods	*A. fumigatus*	*A. fumigatus*	*A. fumigatus*	*A. quadrilineatus*	*A. quadrilineatus*
**Treatment and outcome**					
Treatment	Amphotericin B	Amphotericin B	Micafungin	Amphotericin B and voriconazole	Amphotericin B
Outcome	Died; death attributed in part to aspergillosis	Recovered	Recovered	Recovered	Recovered

**Abbreviation:** NICU = neonatal intensive care unit.

* Extremely preterm neonates are infants who are born before 28 weeks of gestation. Extremely low birthweight = <1,000 g (<2.2 lbs).

### New York NICU

During July 2024, clinicians at a level IV New York NICU identified an open skin lesion on the back of an extremely preterm neonate (GA = 24–25 weeks) who was receiving invasive mechanical ventilation, during the first week of life (Table); culture grew *A. fumigatus*. One month later, a second extremely preterm neonate (GA = 24–25 weeks) was noted to have an excoriated lesion during the first week of life; culture obtained at onset grew a non-*fumigatus Aspergillus* species. In September 2024, a third extremely preterm neonate (GA <24 weeks) developed raised lesions on the back during the second week of life; culture grew a non-*fumigatus Aspergillus* species. The first, second, and third New York patients were treated with micafungin, voriconazole plus amphotericin B, and amphotericin B, respectively; all survived. State public health laboratory testing confirmed the first isolate as *A*. *fumigatus* and reclassified the second and third isolates as *Aspergillus quadrilineatus* by ITS sequencing and BLAST search. Whole-genome sequencing suggested that the two *A*. *quadrilineatus* isolates were closely related. The New York facility reinforced skin checks and conducted an assessment of the NICU environmental and surrounding areas. No specific areas of concern or recent nearby construction were identified; environmental testing was not conducted. Mitigation measures included performing additional linen-dryness checks (e.g., inspecting for dampness or residual moisture, condensation, or stains), replacing some laundered linens with sterile towels, implementing use of disposable positioning devices for extremely preterm neonates, and requiring sterile gloves during care of NICU neonates during their first 4 days of life. The facility also provided chlorhexidine-gluconate–impregnated surgical scrubs for staff members and visitors, reviewed disinfectant products and sanitation procedures, inspected the air-handling system to ensure appropriate pressure in linen-storage areas, and proactively replaced high-efficiency particulate air filters. As of September 1, 2026, no additional cases had been identified in either NICU.

## Discussion

These cases highlight the risk for cutaneous aspergillosis among extremely preterm neonates and the importance of early recognition and testing of suspicious skin lesions. In both NICUs, recognition of cases prompted enhanced surveillance; multidisciplinary assessments of infection prevention practices by infection preventionists, clinicians, facility engineers, and public health staff members; and implementation of mitigation efforts, despite the absence of an identified environmental source. Because environmental molds are ubiquitous and health care exposures can occur through multiple pathways (e.g., construction, contaminated textiles, or medical equipment), environmental sampling and testing might be of limited value unless guided by a specific epidemiologic hypothesis. Interpretation of environmental sampling is further limited by the absence of baseline comparison data, and sampling might be more useful in facilities with established baseline measurements or continuous air monitoring. It is important that mitigation not be delayed while awaiting identification of a definitive environmental source (*2*).

### Limitations

The findings in this report are subject to at least three limitations. First, as is common among health care–associated mold outbreak investigations (*2*), no definitive environmental source was identified in either cluster. Second, requiring a positive skin-lesion culture might have led to undercounting, because variation in culture timing and yield can preclude case identification. Finally, multiple mitigation measures were implemented simultaneously, preventing evaluation of the effectiveness of specific interventions.

### Implications for Public Health Practice

Clinicians and infection preventionists should consider cutaneous aspergillosis when evaluating new erythematous, ulcerative, necrotic, or otherwise suspicious skin lesions among extremely preterm neonates, who can have fragile skin barriers and frequent exposure to invasive medical devices (*1*). Prompt culture or biopsy of suspicious lesions, species-level identification and assessment of relatedness when available, and coordination among clinicians, infection preventionists, facility engineers, environmental services, public health agencies, and laboratories can support timely case finding, environmental assessment, and risk reduction. Mold infection risks and prevention strategies can be included in ongoing education activities for NICU staff members and caregivers. CDC tools and guidance can support assessment of environmental sources,** outbreak response and mitigation,^††^ infection prevention practices,^§§^ and laundry handling.^¶¶^

## Footnotes

* These authors contributed equally to this report.

† Extremely preterm neonates are infants who are born before 28 weeks of gestation.

§ Level IV is the highest level of neonatal care, providing advanced neonatal intensive care for critically ill infants.

¶ 45 C.F.R. part 46, 21 C.F.R. part 56; 42 U.S.C. Sect. 241(d); 5 U.S.C. Sect. 552a; 44 U.S.C. Sect. 3501 et seq.

** CDC’s Targeted Environmental Investigation Checklist for environmental fungi provides a structured framework for assessing potential health care–associated mold sources, including air handling, water intrusion, construction, cleaning, and patient-care materials.

†† CDC guidance for health care–associated mold outbreaks describes approaches to case finding, environmental assessment, mitigation, and coordination with public health and laboratory partners.

§§ CDC’s Infection Control Assessment and Response tool supports systematic review of health care infection prevention practices, including environmental cleaning, hand hygiene, personal protective equipment, and facility-level practices including a supplemental module focused on NICU-specific infection prevention practices.

¶¶ A laundry facility checklist provides a structured approach for reviewing health care textile handling, transport, storage, laundering, and separation of clean and soiled linens. Infection Control Assessment of Laundry Facility Preparedness to Keep Healthcare Textiles Clean
